# Self-esteem as a catalyst for change in adolescent inpatients with anorexia nervosa: a pilot randomised controlled trial

**DOI:** 10.1007/s40519-021-01161-0

**Published:** 2021-03-13

**Authors:** Hannah Biney, Emma Giles, Matt Hutt, Rachel Matthews, J. Hubert Lacey

**Affiliations:** 1 Schoen Clinic Newbridge, Birmingham, UK; 2 St George’s, University of London, London, UK

**Keywords:** Self-esteem, CBT, Adolescents, Anorexia nervosa, Group therapy

## Abstract

**Purpose:**

To determine the potential effectiveness of a six-session manualised self-esteem group using CBT approaches when given as an adjuvant to adolescent inpatients with Anorexia Nervosa (AN).

**Methods:**

Using a randomised controlled design, 50 girls aged 12–17 years with AN were assigned to either self-esteem group with treatment as usual (TAU) (*n* = 25) or TAU alone (*n* = 25). 50/78 (64%) consented to be randomised. Both groups completed self-report measures of self-esteem and eating disorder psychopathology at three time points to measure the potential effectiveness of the treatment. Qualitative feedback was collected to assess acceptability.

**Results:**

29 participants completed the study: 15 self-esteem group with TAU, 14 TAU alone. Self-esteem group participants had greater improvement in all outcomes than TAU participants at all time points, the difference in self-report self-esteem at T2 is 1.12 (95% CI − 1.44–3.69; effect size = 0.21). Similar small effect sizes were found for the eating disorder psychopathology measure following completion of the intervention but not at four-week follow-up. Favourable qualitative feedback was gained.

**Conclusion:**

These findings demonstrate that the self-esteem group supplements an intensive treatment package which also addresses elements of low self-esteem. The self-esteem group was beneficial for addressing self-esteem and acted as a catalyst for change in eating disorder psychopathology. Positive qualitative feedback indicated the intervention was acceptable to users. Self-esteem group is a potential new adjuvant treatment for AN.

**EMB Rating:**

Level 1.

**Supplementary Information:**

The online version contains supplementary material available at 10.1007/s40519-021-01161-0.

## Introduction and aims

Self-esteem is the extent to which we like and value ourselves, including confidence and acceptance of our worth and abilities [1]. Individuals with low self-esteem have a negatively biased view of themselves, with a strong focus on their flaws, weaknesses and mistakes, which tend to be global, persistent and enduring [1]. Healthy self-esteem is an important factor in the development and maintenance of good psychological wellbeing [2]. Low self-esteem is a core feature in a variety of mental health problems [3], and it has been found to be amongst the best predictors of emotional and behavioural difficulties [4]. Although Cognitive Behavioural Therapy (CBT) for low self-esteem is commonly used in clinical practice, very few studies have focused on self-esteem targeted treatments in comparison to the evidence base for CBT interventions for mood disorders, such as depression and anxiety [5–9].

Low self-esteem has been identified as a strong predictor of onset, maintenance and relapse in eating disorders [10–14]. It is well evidenced in the literature that individuals with Anorexia Nervosa (AN) have reduced self-esteem in comparison to non-eating disordered controls [12, 15–18]. For patients with low self-esteem, psychological change can be particularly difficult to achieve, with the potential for treatment drop-out, prolonged admissions and increased burden of illness to the individual and their family or carers al., [19]. Furthermore, low self-esteem can serve to maintain pervasive negative self-regard and a sense of little or no prospect of recovery. Button and Warren [20] found that difficulties with low self-esteem persisted following weight restoration and improvements in eating behaviours.

Patients with significant low self-esteem often strive to gain a sense of self-worth by controlling eating, weight and shape. This has been supported by Brockmeyer et al. [21] who suggested that in AN, self-esteem is associated with low weight, which is viewed as an achievement. This may undermine motivation to engage in positive change since weight gain would require the individual to relinquish the achievement, thus lowering self-esteem. This indicates that supporting individuals to value other, none weight and shape related aspects of themselves could support motivation to change. These findings suggest treatment of low self-esteem is clinically important. Inpatient treatment for AN is expensive for health care systems [22]; any treatment which improves the likelihood of recovery should be evaluated.

The direction of causality between low self-esteem and eating disorder psychopathology is unclear. There is evidence arguing that low self-esteem occurs prior to the onset of the eating disorder, therefore eating disorders can be viewed as a symptom of low self-esteem [14, 23, 24]. Interventions which target self-esteem have shown considerable potential for reducing eating disorder psychopathology [23].

Individuals with AN and their carers have both stated the importance of targeting self-esteem during the treatment of AN [25]. Several studies have therefore explored change in self-esteem following provision of a specialist inpatient treatment programme. Karpowicz et al. [24] evidenced improved self-esteem, body mass index (BMI), weight phobia and body dissatisfaction following three months of specialist inpatient treatment in 16–25 year olds with AN. This treatment consisted of support from a multidisciplinary team (MDT) where a cognitive approach was used to support the patients in exploring new ways of managing problematic thinking patterns and behaviours. Furthermore, Collin et al. [26] evidenced improvements in self-esteem with eating disorder psychopathology and BMI over the course of an inpatient treatment programme for women with AN. This study used a multi-dimensional measure of self-esteem and found that different domains of self-esteem had unique implications for eating disorder psychopathology. Lovability and moral self-approval at baseline were predictive of change in shape and weight concern, whereas change in self-control and lovability were predictive of change in dietary restraint and shape concern. These findings highlight the complex relationship between self-esteem and eating disorder psychopathology.

Further research has used specific interventions targeted at improving low self-esteem, in particular CBT-based group interventions. Fleming, Doris and Tchanturia [27] designed and piloted a six-week self-esteem group within an inpatient adult eating disorders ward. The findings were mixed; qualitative feedback demonstrated that the group was acceptable and useful, however, no statistically significant improvements in self-esteem were found, although a trend in the positive direction was observed. Furthermore, Newns et al., [23] found that a long-term intervention over the course of 20 months led to improved self-esteem in eating disordered (though not exclusively AN) participants. Further evidence for a brief, CBT-based self-esteem group therapy comes from Adamson, Ozenc, Baille and Tchanturia, [28] who found that a six-week group led to significant improvements in self-esteem and a small improvement in self-perceived ability to change in women with AN in an inpatient setting. These findings identify that a group-based approach to improving low self-esteem in adult populations is effective and further research is required exploring this in an adolescent population.

To our knowledge, research into the effects of a self-esteem CBT group intervention with adolescents is scarce. Lázaro et al. [29] found that a CBT-based self-esteem and social skills group adolescents with AN-related disorders improved perceptions of appearance, happiness, satisfaction, social withdrawal, and leadership. Furthermore, the authors of this paper reported significant improvement in self-esteem following a six-week CBT-based group for adolescents with AN in an inpatient setting [30]. These findings were maintained at 4-week follow-up and positive qualitative feedback indicated the acceptability of the intervention; however, a control group was not included. These studies indicated the need for future controlled studies; therefore, the aim of the current study was to replicate these findings utilising a treatment as usual (TAU) control group.

Fennel’s [31] model proposes that low self-esteem develops from negative life experiences, particularly in early life that influences our thoughts, beliefs, attitudes and opinions about self which serve to maintain low self-esteem. Based on this model Fennel has produced guided self-help books which can be used to understand how low self-esteem develops and is maintained. To improve low self-esteem, techniques for questioning and challenging these cognitions are required. This model of low self-esteem and the self-help book, Overcoming Low Self-esteem: a self-help guide using cognitive behavioral techniques [32] formed the basis of our group intervention. By using a group format, we hoped that the young people would also benefit from a positive experience of being accepted and valued by their peers, in addition to opportunities to learn from others and improve communication skills [33].

The aim of this study was to evaluate the potential effectiveness of the self-esteem group which uses CBT approaches to improve self-esteem in adolescents with a diagnosis of AN, when used as an adjuvant to an array of therapies in an inpatient unit [34, 35, 36]. Qualitative feedback from participants in the intervention group was also sought to evaluate the acceptability and experience of the intervention. The feasibility of a full trial is also explored.

## Materials and methods

### Participants

Participants were child and adolescent girls with a primary diagnosis of AN currently receiving inpatient treatment at Newbridge House. The diagnosis was established at admission using DSM-5 criteria [37]. In the UK children and adolescents with AN are typically treated as outpatients. The patients referred to Newbridge House were referred by the NHS because their local psychiatrists deemed them to be severely ill and could not be managed safely in the community at home.

Participants were recruited for the study as they were approaching a median BMI of 85%. This weight criterion ensured participants had sufficient time to complete the study before discharge.

Inclusion criteria included a primary diagnosis of AN, aged between 11–18 and currently receiving inpatient treatment at Newbridge House. Exclusion criteria included previously completing the self-esteem group at Newbridge House during the therapy’s development stage, moderate and severe learning difficulty, and being aged under 10 years. Inclusion and exclusion criteria were assessed using clinical documentation or through discussions with the MDT.

A sample size of 50 was considered acceptable to address the objectives of this study. By randomising 25 participants into each treatment arm differences between groups, with respect to self-reported self-esteem and self-reported eating disorder psychopathology, could be estimated with 95% confidence intervals.

Prior written informed consent was gained from all patients and their parents. The consenting procedure and forms were agreed and authorised by the West Midlands-Black Country NHS Ethics Committee. The study was reviewed by the Newbridge Research and Ethics Committee. IRAS project ID: 234036.

### Self-esteem group therapy

The self-esteem group is a six-session manualised programme developed at Newbridge House for adolescents based on Melanie Fennel’s model for low self-esteem in adults [31, 32]. The intervention includes similar concepts to the core low self-esteem module which is incorporated in the CBT for eating disorders manual [11].

This intervention is an educational and therapeutic group which aims to provide psychoeducation focusing on issues relating to low self-esteem, where it comes from and how it can be affected. The group adopts a CBT approach to support low self-esteem; including the use of three and five column thought records, behavioural experiments and positive data logging. Each session was an hour long and started with a check-in and homework review. It is expected that work is completed outside of sessions and that techniques are put into practice to ensure maximum benefit from the group.

See Table 1 for details of the programme structure. The manual is available on request from the corresponding author.

**Table 1:** Programme structure

Session number	Session outline	Homework tasks
1	Introduction to the group Exploration of self-esteem and self-confidence Introduction of the CBT model CBT works on the basis that our thoughts effect how we feel and in turn effect our behaviour	Antecedent-Behaviour-Consequence diary to identify thinking patterns, associated emotions and behaviours
2	Exploration of how low self-esteem can develop from a variety of negative early experiences Develop self-esteem formulation Formulation is the process of making sense of a person’s difficulties in the context of their relationships, social circumstances, life events and the meaning they have made of these experiences	Reflection on formulation and impact of low self-esteem
3	Introduction to negative automatic thoughts and thought biases Negative automatic thoughts influence the way an individual see’s themselves, others and their future Thought biases are faulty patterns of thinking that are self-defeating and often don’t match with reality Three column thoughts records Three column thought records identify situations, emotions and negative automatic thoughts and are used as a first step to the process of cognitive restructuring in CBT Problem solving and distraction techniques	Three column thought records Problem solving activity
4	Introduction to hot thoughts The hot thought is the negative automatic thought that causes the most distress and influences our feelings and behaviours the most Five column thought records Five column thought records identify situation, emotion, negative automatic thoughts, alternative response and outcomes and are used to support cognitive restructuring in CBT	Five column thought records
5	Introduction to rules for living Rules for living guide our behaviour and enable us to cope with our everyday lives Setting up behavioural experiments Behavioural experiments are an information gathering exercise, which is used to test the accuracy of an individual’s beliefs or to test out more adaptive beliefs	Behavioural experiment
6	Increasing self-acceptance and acknowledging positive qualities Positive data logging Positive data logging is a record of positive experiences to support development of more adaptive ways of thinking	Positive data logging

### Treatment as usual

TAU refers to the standard inpatient treatment programme at Newbridge House which includes individual and group support with Occupational Therapists, Dieticians, Nurses, Psychologists and Psychiatrists and a leisure programme. Some of these treatments were practical dealing with meals or food preparation and others were psychological addressing body image, self-esteem or family therapy. Three novel treatments from the hospital have previously been published [34–36]. Additionally, some groups were psychoeducational. Medication is rarely used and always briefly, details have not been included in this research project. All treatments took place around the in-house school teaching programme which maintained the children’s education. Details of all these activities can be found on the Newbridge House website.

### Measures

#### Rosenberg self-esteem scale (RSE) [35]

The RSE is a ten item self-report measure of self-esteem. The RSE asks individuals to consider how much they agree with ten statements on a four-point scale from ‘strongly agree’ to ‘strongly disagree’. Higher scores on the RSE indicates higher self-esteem. A score of 21 or above indicates healthy self-esteem. The RSE has shown good reliability and validity across a range of sample groups, including adolescents [39].

### Eating disorder examination questionnaire (EDE-Q) [40]

The EDE-Q is a 28 item self-report measure developed from the eating disorder examination (EDE) interview-based assessment tool [41], which assesses the core psychopathology of eating disorders. It includes four subscales: restraint, eating concern, shape concern and weight concern. The EDE-Q asks individuals to answer questions based on the previous four weeks on a six-point scale. Higher scores on the EDE-Q indicate greater eating disorder psychopathology. Internal consistency is good with Cronbach’s alpha coefficients ranging from 0.70 to 0.83 in a clinical sample [39].

### The feedback form

Qualitative feedback was collected following completion of the programme. It included open-ended questions asking the young people “what did they find most helpful?”, “was there anything about the sessions that was unhelpful?” and “how would you improve the programme?”

### Procedure

Patients approaching a median BMI of 85% were assessed by the Research Team at Newbridge House for suitability for the study using the framework of inclusion and exclusion criteria. The Research Team also work clinically as Assistant Psychologists. Those meeting inclusion criteria were then approached by an Assistant Psychologist who introduced the research, provided an information sheet about the study and sought informed consent for participation. Both parents and patients were given seven days to consent to the research.

Patients for whom we received appropriate consent were then allocated to a treatment or control group, where treatment involved receiving self-esteem group therapy as well as TAU and control involved TAU only. The block randomisation method was used to randomise subjects into groups resulting in equal sample sizes across the treatment and control groups. All participants completed baseline self-esteem and eating disorder psychopathology measures (T1) after which those in the treatment group commenced self-esteem group therapy and those in the control group continued to receive TAU. After completion of the group (week 6) all participants completed a second set of measures (T2). Four weeks post-completion of the group (week 10) all participants then completed a final set of measures (T3).

### Statistical analyses

This was a pilot study. The primary aim of the data analysis phase was not to definitively test whether the self-esteem group therapy is effective or not but to generate evidence of its potential effectiveness if progressed into a full trial. Analysis of covariance (ANCOVA) was used to estimate the difference in self-reported self-esteem at week 6 (T2) and week 10 (T3) between conditions, controlling for baseline (T1) self-reported self-esteem as a covariate. The difference in mean self-reported self-esteem scores is reported (adjusted for baseline levels) with 95% confidence intervals to indicate the potential effectiveness of the self-esteem group therapy intervention in comparison to TAU. The assumptions of normality and homogeneity of variance were assessed using the One-Sample Kolmogorov–Smirnov test and the Levene’s Test for the Equality of Error Variances. This analysis was repeated for the self-reported eating disorder psychopathology outcome.

In addition, to evaluate clinical significance, Cohen’s D [43] was calculated to provide effect sizes, with an effect size of 0.2 defined as small, 0.5 defined as medium and 0.8 defined as large.

## Results

### Participant characteristics/descriptive statistics

86 child and adolescent girls with AN were assessed for eligibility to take part in the study; eight (9%) were excluded due to not meeting the inclusion criteria, 28 (33%) declined to participate and 50 (58%) consented. Fifty child and adolescent girls were recruited and randomised to the study. The mean age of the sample was 15.22 years (SD = 1.62) ranging from 12 to 17. Four of the 50 (8%) had a comorbid diagnosis of Autistic Spectrum Disorder (ASD). In the intervention group, 16 (64%) were receiving CBT-E, seven (28%) were receiving psychodynamic psychotherapy, one (4%) had some sessions of CBT and then discontinued and one (4%) declined to attend individual therapy. In the control group, 17 (68%) were receiving CBT-E and eight (32%) were receiving psychodynamic psychotherapy. This highlights that there was a relatively even distribution of therapeutic modality across intervention groups. The mean duration of admission prior to starting the research was 70.48 days (SD = 28.55). Weight for height and BMI was collected for all participants at all time points (see Table 2).

**Table 2:** Mean weight for height and mean BMI at T1, T2 and T3

Time point	TAU				Self-esteem + TAU			
	Mean weight for height	SD	Mean BMI	SD	Mean weight for height	SD	Mean BMI	**SD**
1	90.86	6.67	18.26	1.28	90.56	5.74	18.20	1.43
2	96.38	4.88	19.40	1.04	96.60	3.79	19.46	1.14
3	97.92	3.98	19.84	0.73	97.23	3.05	19.66	0.76

Twenty-five participants were randomised to the self-esteem group condition and 25 to the control condition. Twenty-one young people did not complete the study. Four young people dropped out of the study prior to T1, five young people dropped out of the study prior to T2 and a further 12 young people dropped out of the study prior to T3. Reasons for non-completion of the study included: team discharge prior to completion of research (*n* = 17), discharged against medical advice (*n* = 1) and withdrew consent from the research project (*n* = 3). The CONSORT diagram (see supplementary materials) provides further information related to drop out rates by randomised group. Following discharge from inpatient services, participants are not followed up for research purposes given the large changes in environmental factors and treatment between inpatient and community settings which would likely influence the results. Therefore, 41 participants completed the study up to T2: 21 in the self-esteem group and 20 in TAU. Additionally, 29 participants completed the study up to T3: 15 in the self-esteem group and 14 in TAU. These participants make up the dataset for the two sets of analyses.

Normality and homogeneity of variance were indicated, results not given here.

### Changes in self-reported self-esteem following self-esteem group therapy

Mean self-reported self-esteem scores were calculated for the control and treatment group at the start (T1) and end of the programme (T2), see Table 3. Mean scores increase in both conditions between T1 and T2. The difference in mean scores at T2 (controlling for baseline outcome) indicate better self-reported self-esteem in the treatment group, difference in means = 1.12 (95% CI: − 1.44, 3.69). A positive difference in means indicates the self-esteem group intervention having a greater effect than the control. A small effect size was noted (Cohen’s *D* = 0.21).

**Table 3:** Comparison of conditions with respect to self-reported self-esteem and eating disorder psychopathology outcomes at T2, difference in means (Self-esteem + TAU vs TAU, controlling for baseline outcome), 95% confidence intervals (CI) and Cohen’s D

Measures	TAU (*N* = 20)				Self-esteem + TAU (*N* = 21)				Mean difference (95% CI)	Cohen’s D
	T1		T2		T1		T2			
	Mean	SD	Mean	SD	Mean	SD	Mean	SD		
RSE	18.80	4.56	20.05	5.58	18.29	4.03	21.19	5.01	1.12 (− 1.44, 3.69)	0.21
EDE-Q: weight concern	4.08	1.82	3.77	2.38	4.29	1.62	3.17	1.94	0.43 (− 0.36, 1.21)	0.28
EDE-Q: shape concern	4.89	1.32	4.33	2.28	4.95	1.38	3.97	1.76	0.74 (− 0.10, 1.58)	0.17
EDE-Q: eating concern	2.84	1.73	2.68	1.87	3.19	1.58	2.28	1.60	0.66 (0.44, 1.28)	0.23
EDE-Q: dietary restraint	2.76	2.36	3.08	2.59	3.51	2.36	2.51	2.26	1.12 (− 0.00, 2.23)	0.23
EDE-Q: global score	3.64	1.68	3.46	2.20	3.97	1.63	2.98	1.79	0.81 (0.08, 1.54)	0.24

*RSE* Rosenberg self-esteem scale, *EDE-Q* eating disorders examination questionnaire

Mean self-reported self-esteem scores were also calculated for the control and treatment group at the four-week follow-up (T3), see Table 4. Mean scores increase in both conditions between T1 and T3. The difference in mean scores at T3 (controlling for baseline outcome) indicate marginally better self-reported self-esteem in the treatment group in comparison to the control group. Negligible effect sizes were noted (Cohen’s *D* = 0.04).

**Table 4:** Comparison of conditions with respect to self-reported self-esteem and eating disorder psychopathology outcomes at T3, difference in means (Self-esteem + TAU vs TAU, controlling for baseline outcome), 95% confidence intervals (CI) and Cohen’s D

Measures	TAU (*N* = 14)				Self-esteem + TAU (*N* = 15)				Mean difference (95% CI)	Cohen’s D
	T1		T3		T1		T3			
	Mean	SD	Mean	SD	Mean	SD	Mean	SD		
RSE	17.86	4.61	21.14	7.17	18.00	3.95	21.40	4.91	0.15 (− 4.10, 3.82)	0.04
EDEQ: Weight Concern	4.46	1.74	3.67	2.00	4.43	1.66	3.43	2.02	0.22 (− 0.90, 1.34)	0.11
EDE-Q: shape concern	5.10	1.33	4.20	2.07	5.10	1.44	4.18	1.94	0.02 (− 1.18, 1.21)	0.01
EDE-Q: eating concern	3.20	1.53	2.53	1.61	3.89	1.70	2.47	1.74	0.19 (− 0.77, 1.16)	0.04
EDE-Q: dietary restraint	3.11	2.38	2.71	2.43	3.88	2.39	2.75	2.12	0.53 (− 0.63, 1.69)	0.02
EDE-Q: global score	3.97	1.60	3.28	1.94	4.20	1.67	3.20	1.85	0.27 (− 0.75, 1.28)	0.04

*RSE* Rosenberg self-esteem scale, *EDE-Q* eating disorders examination questionnaire

### Changes in self-reported eating disorder psychopathology following self-esteem group therapy

Mean self-reported eating disorder psychopathology scores were calculated for the control and treatment groups at the start (T1) and end of the programme (T2), see Table 3. Mean scores decrease in both conditions between T1 and T2. The difference in mean scores at T2 (controlling for baseline outcome) indicate reduced eating disorder psychopathology in the treatment group in comparison to the control group for all outcomes. A positive difference in means indicates the self-esteem group intervention having a greater effect than the control. Small effect sizes were noted ranging from 0.17 for shape concern to 0.28 for weight concern. The 95% confidence intervals indicate that there is a significant difference in global eating disorder psychopathology, eating concern [and dietary restraint between the treatment and control groups at T2.

Mean self-reported eating disorder psychopathology scores were also calculated for the control and treatment group at the four-week follow up (T3), see Table 4. Mean scores decrease in both conditions between T1 and T3. The difference in mean scores at T3 (controlling for baseline outcome) indicated reduced eating disorder psychopathology in the treatment group in comparison to the control group for all outcomes except for dietary restraint. Negligible effect sizes were noted ranging from 0.02 for dietary restraint to 0.11 for weight concern.

### Evaluation form

Feedback showed the treatment was acceptable to users. The patients reported that the sessions were helpful in understanding what self-esteem was and the impact it was having on their functioning. “The group provided useful tips and good life skills”. “The use of case studies was helpful to support my understanding”. Other themes included learning how self-esteem develops, exploring ways to challenge negative thoughts and feeling listened to. Session six concerning self-acceptance and acknowledging positives was reported to be the most helpful. “I have learnt that it is okay to be myself”. There were a few negative comments including “feeling pressure to complete monitoring records” and there being “too much practical work.” Some patients made suggestions for improvements such as “having more videos and interactive tasks” and “completing more personal tasks.”

## Discussion

To the author’s knowledge, this is the first RCT study to provide evidence of a brief manualised group intervention targeting low self-esteem, designed for children and adolescents with AN. This is in accordance with NICE guidelines [44] which welcome contributions of brief inpatient, evidence-based treatment protocols to clinical service provision. The study reports on the effectiveness and acceptability of the self-esteem group therapy when used as an adjuvant therapy in an inpatient unit. It also investigates the therapeutic benefit of the programme in reducing eating disorder psychopathology. Effectiveness was measured by self-report questionnaires and acceptability was assessed using qualitative data obtained from participant’s feedback.

Effect sizes suggest the self-esteem group therapy was beneficial for addressing low self-esteem. Furthermore, the magnitude of the effect sizes on the RSE (Cohen’s *D* = 0.21) are comparable to previous studies that have reported effect sizes on the same measure following self-esteem intervention (Cohen’s *D* = 0.13–0.39) [27, 28]. The Treatment as Usual programme at Newbridge House has been rated independently by the UK government’s Care Quality Commission as “Outstanding” [45]. The clinical effects of this treatment should be judged against this. The authors believe that the programme could be of clinical importance and a potential new treatment for AN, certainly if offered in a setting that was not so therapeutically dense [34–36] (Treatment as usual for above). Therefore, the programme may be more beneficial in an outpatient setting which typically offers fewer therapeutic interventions. Furthermore, the effect sizes provide evidence that the intervention should be tested in a larger scale RCT which could be extended into an outpatient setting whereby a larger sample size would be available.

These findings add further support to previous research showing the effectiveness of CBT-based interventions for the treatment of low self-esteem for adults [23, 27, 28] and adolescents [29, 30] with AN or AN-related disorders.

This study also explored the influence of self-esteem group therapy on eating disorder psychopathology. Self-esteem group therapy produced significant changes in self-reported eating disorder psychopathology, in particular global eating disorder psychopathology, dietary restraint and eating concern. Furthermore, effect sizes suggest the self-esteem group therapy was beneficial for addressing eating disorder psychopathology. These findings suggest that interventions that target self-esteem can act as a catalyst for change in eating disorder psychopathology. This is supported by Newns et al., [23] who evidenced interventions for self-esteem have considerable potential for improving eating disordered attitudes.

These findings also add further support to previous research highlighting that specialist inpatient treatment programmes can improve both self-esteem and eating disorder psychopathology [24, 26]. To the authors knowledge, this is the first controlled study which has found a significant improvement in eating disorder psychopathology following intervention for low self-esteem.

It is not surprising that greater changes are seen in weight and eating disorder psychopathology, than in self-esteem which is seen as a stable personality trait. Surgenor, Maguire, Russell and Touyz, [46] found that one in three participants reported a reduction in self-esteem over treatment despite improvements in BMI and eating disorder psychopathology. Furthermore, Button and Warren [20] evidenced that difficulties with low self-esteem persisted following weight restoration and improvements in eating behaviours. An improvement in weight and eating disorder behaviours is not always viewed positively by patients [47, 48] and often low weight leads to increased self-esteem due to experiencing weight loss as a success [21]. This suggests that improvements in self-esteem may be dependent on the individual and their views towards recovery.

The qualitative feedback gained from the participants following completion of the self-esteem group was positive and indicated that the intervention was acceptable to users. A number of individuals reported improvements related to supporting their understanding of low self-esteem and gaining psychological skills to improve their self-esteem and self-acceptance.

This study included a 4 weeks follow-up (T3) to explore the stability of intervention effects. Although improvements in self-esteem and eating disorder psychopathology were found for the treatment group, differences between the treatment and control group were minimal. These results may be explained by the attrition rate between T2 (*n* = 41) and T3 (*n* = 29). All individuals who dropped out of the research between T2 and T3 had been discharged by the clinical team because they had achieved a maintenance period at a healthy weight and had completed the therapeutic programme; the drop-out was not due to the self-esteem treatment. Furthermore, individuals often completed this measure in the final weeks prior to discharge when they are re-integrating back into the community, including spending more time in their family home and attending their home school. Adolescents with AN have explained that they felt “removed from the outside world” and that there was a “high level of structure and support” whilst they were in an inpatient setting which created difficulties for readjustment during the transition and discharge process [49].

Research within the field of AN, and in inpatient settings in particular, is challenging, all the more so, when the treatment is tested in an active unit providing a dense treatment programme to public health funded patients. The patients’ ambivalence regarding recovery, clinical staff priorities and retention of participants due to variable admission lengths are well recognised problems [50]. The number of patients who were discharged prior to completion of the research highlights the difficulty of implementing group therapeutic interventions in inpatient settings with relatively short admissions and raises the question of how to evaluate these effectively. Consideration of whether participants can remain in the study following discharge would enable retention of sample size but may likely confound the results given the stark differences between inpatient and community settings. The authors also recognise the inherent biases associated with investigating the effectiveness of an intervention that they were involved in developing and facilitating. Future research would benefit from independent researchers replicating these findings. Furthermore, future research may also benefit from utilising a multi-dimensional measure of self-esteem, given recent findings of differential associations between domains of self-esteem and eating disorder psychopathology [26].

Low self-esteem is widely acknowledged as a core feature in a variety of mental health problems [3] and has been identified as a strong predictor of onset, maintenance and relapse in eating disorders [10–14]. Furthermore, individuals with AN and their carers have both stated the importance of targeting self-esteem during the treatment of AN [25]. However, there is a limited evidence base regarding the effectiveness of interventions targeting low self-esteem, particularly for adolescents with AN. This study explored the effectiveness of a novel, manualised, brief group intervention for low self-esteem. Overall, the findings suggest a specific self-esteem intervention leads to significant improvements in eating disorder psychopathology and does not detract from an intensive treatment programme. These results are particularly reassuring because the self-esteem group therapy was tested against TAU which included a battery of standardised treatments, including individual and group therapy, exercise and activity groups, occupational therapy, family therapy, dietetic counselling, nursing support, schooling and medication prescribed by a Consultant Psychiatrist. CBT threads were in both the individual and group work. Details can be found on the Newbridge House Website and references [34–36].

These results suggest that the programme is of clinical importance and that targeting self-esteem is an effective catalyst for change in eating disorder psychopathology. As well as adding to the existing literature, it is hoped that this study encourages further controlled research into the use of CBT-based interventions for self-esteem for adolescents with AN as the current literature is scarce.

### What is already known on this subject?

Uncontrolled studies of CBT-based group therapies evidenced improved self-esteem in adult women and adolescents with Anorexia Nervosa (AN) in both inpatient and outpatient settings.

### What this study adds?

A randomised controlled trial of a self-esteem group therapy, using CBT is evidenced as an effective catalyst for change in self-esteem and eating disorder psychopathology for adolescents with AN.

## Supplementary Information

Below is the link to the electronic supplementary material.

Supplementary file1 (DOCX 73 KB)

## Data Availability

The data sets generated during and/or analysed for the current study are available from the corresponding author on reasonable request. The Manual is available from the corresponding author.

## References

[1] Fennell MJ (1997). Low self-esteem: a cognitive perspective. Behav Cogn Psychother.

[2] Morton L, Roach L, Reid H, Stewart SH (2012). An evaluation of a CBT group for women with low self-esteem. Behav Cogn Psychother.

[3] Schaefer CE, Drewes AA (2013). The therapeutic powers of play: 20 core agents of change.

[4] Silverstone PH, Salsali M (2003). Low self-esteem and psychiatric patients: part I—the relationship between low self-esteem and psychiatric diagnosis. Ann Gen Hosp Psychiatry.

[5] Covin R, Ouimet AJ, Seeds PM, Dozois DJ (2008). A meta-analysis of CBT for pathological worry among clients with GAD. J Anxiety Disord.

[6] Gaffan EA, Tsaousis J, Kemp-Wheeler SM (1995). Researcher allegiance and meta-analysis: the case of cognitive therapy for depression. J Consult Clin Psychol.

[7] Robinson LA, Berman JS, Neimeyer RA (1990) Psychotherapy for the treatment of depression: a comprehensive review of controlled outcome research. Psychol Bull 108:30. Retrieved from: https://ebbp.org/resources/Depression_Berman.pdf 10.1037/0033-2909.108.1.30 2200072

[8] Westen D, Morrison K (2001). A multidimensional meta-analysis of treatments for depression, panic, and generalized anxiety disorder: an empirical examination of the status of empirically supported therapies. J Consult Clin Psychol.

[9] Whitfield G, Williams C (2003). The evidence base for cognitive—behavioural therapy in depression: delivery in busy clinical settings. Adv Psychiatr Treat.

[10] Button EJ, Sonuga-Barke EJS, Davies J, Thompson M (1996). A prospective study of self-esteem in the prediction of eating problems in adolescent schoolgirls: questionnaire findings. Br J Clin Psychol.

[11] Fairburn CG (2008). Cognitive behavior therapy and eating disorders.

[12] Fairburn CG, Cooper Z, Shafran R (2003). Cognitive behaviour therapy for eating disorders: a “transdiagnostic” theory and treatment. Behav Res Ther.

[13] Fairburn CG, Peveler RC, Jones R, Hope RA, Doll HA (1993). Predictors of 12 month outcome in bulimia nervosa and the influence of attitudes to shape and weight. J Consult Clin Psychol.

[14] Silverstone PH (1992). Is chronic low self-esteem the cause of eating disorders?. Med Hypotheses.

[15] Cervera S, Lahortiga F, Angel Martínez-González M, Gual P, Irala-Estévez JD, Alonso Y (2003). Neuroticism and low self-esteem as risk factors for incident eating disorders in a prospective cohort study. Int J Eat Disord.

[16] Jacobi F, Wittchen HU, Hölting C, Höfler M, Pfister H, Müller N, Lieb R (2004). Prevalence, co-morbidity and correlates of mental disorders in the general population: results from the German Health Interview and Examination Survey (GHS). Psychol Med.

[17] Silverstone PH (1990). Low self-esteem in eating disordered patients in the absence of depression. Psychol Rep.

[18] Wilksch S, Wade TD (2004). Differences between women with anorexia nervosa and restrained eaters on shape and weight concerns, self-esteem, and depression. Int J Eat Disord.

[19] Halmi KA, Agras WS, Crow S, Mitchell J, Wilson GT, Bryson SW, Kraemer HC (2005). Predictors of treatment acceptance and completion in anorexia nervosa: implications for future study designs. Arch Gen Psychiatry.

[20] Button EJ, Warren RL (2002). Self-image in anorexia nervosa 7.5 years after initial presentation to a specialized eating disorders service. Eur Eat Disord Rev.

[21] Brockmeyer T, Holtforth MG, Bents H, Kämmerer A, Herzog W, Friederich HC (2013). The thinner the better: self-esteem and low body weight in anorexia nervosa. Clin Psychol Psychother.

[22] Stuhldreher N, Wild B, König HH, Konnopka A, Zipfel S, Herzog W (2015). Determinants of direct and indirect costs in anorexia nervosa. Int J Eat Disord.

[23] Newns K, Bell L, Thomas S (2003). The impact of a self-esteem group for people with eating disorders: an uncontrolled study. Clin Psychol Psychother.

[24] Karpowicz E, Skärsäter I, Nevonen L (2009). Self-esteem in patients treated for anorexia nervosa. Int J Ment Health Nurs.

[25] Vanderlinden J, Buis H, Pieters G, Probst M (2007). Which elements in the treatment of eating disorders are necessary ‘ingredients’ in the recovery process? A comparison between the patient’s and therapist’s view. Eur Eat Disord Rev.

[26] Collin P, Karatzias T, Power K, Howard R, Grierson D, Yellowlees A (2016). Multi-dimensional self-esteem and magnitude of change in the treatment of anorexia nervosa. Psychiatry Res.

[27] Fleming C, Doris E, Tchanturia K (2014). Self-esteem group work for inpatients with anorexia nervosa. Adv Eat Disord.

[28] Adamson J, Ozenc C, Baillie C, Tchanturia K (2019). Self-esteem group: useful intervention for inpatients with anorexia nervosa?. Brain Sci.

[29] Lázaro L, Font E, Moreno E (2011). Effectiveness of self-esteem and social skills group therapy in adolescent eating disorder patients attending a day hospital treatment programme. Eur Eat Disord Rev.

[30] Biney H, Hutt M, Matthews R, Lacey H (2019). An evaluation of a manualised group self-esteem programme for Anorexia Nervosa patients regaining weight. Arch Psychol..

[31] Fennell M (2006). Overcoming low self-esteem self-help course.

[32] Fennell M (2016). Overcoming low self-esteem: a self-help guide using cognitive behavioral techniques.

[33] Tchanturia K (2015). Brief group psychotherapy for eating disorders: inpatient protocols.

[34] Rosewall J, Beavan A, Houlihan C, Bates S, Melhuish L, Mountford V, Lacey JH (2019). Evaluation of teen body wise: a pilot study of a body image group adapted for adolescent inpatients with anorexia nervosa. Eat Weight Disord.

[35] Biney H, Astbury S, Haines A, Grant J, Malone J, Hutt M, Matthews R, Morgan J, White S, Lacey JH (2020). A novel ‘practical body image’ therapy for adolescent inpatients with anorexia nervosa: a randomised controlled trial. Eat Weight Disord.

[36] Mang L, Garghan A, Grant J, Lacey H, Matthews R (2020) An evaluation of efficacy and acceptability of a novel manualised JuniorLEAP group programme for compulsive exercise, for children and adolescents with anorexia nervosa, within an inpatient setting. Eat Weight Disord. 10.1007/s40519-020-00884-w 10.1007/s40519-020-00884-w 32232776

[37] American Psychiatric Association (2013) Diagnostic and statistical manual of mental disorders (DSM-5^®^). American Psychiatric Pub

[38] Rosenberg M (1965) Rosenberg self-esteem scale (RSE). Acceptance and commitment therapy. Measures package 61:18. Retrieved from: http://www.integrativehealthpartners.org/downloads/ACTmeasures.pdf#page=61

[39] Robinson JP, Shaver PR, Wrightsman LS (2013). Measures of personality and social psychological attitudes: measures of social psychological attitudes.

[40] Fairburn CG, Beglin SJ (1994). Assessment of eating disorders: interview or self-report questionnaire?. Int J Eat Disord.

[41] Fairburn CG, Cooper Z, O’Connor M (1993) The eating disorder examination. Int J Eat Disord 6:1–8. Retrieved from https://www.corc.uk.net/media/1274/ede_rcpsychinformation.pdf

[42] Luce KH, Crowther JH (1999). The reliability of the eating disorder examination—self-report questionnaire version (EDE-Q). Int J Eat Disord.

[43] Cohen J (1992). A power primer. Psychol Bull.

[44] National Institute for Health and Care Excellence (2017) Eating disorders: recognition and treatment. NICE guideline NG69. Retrieved from: https://www.nice.org.uk/guidance/ng69 28654225

[45] Care Quality Commission (2018). Newbridge house quality report. https://api.cqc.org.uk/public/v1/reports/cf2f702b-0d79-4bf2-a7e0-61a58b4ad88e?20180731235520

[46] Surgenor LJ, Maguire S, Russell J, Touyz S (2007). Self-liking and self-competence: relationship to symptoms of anorexia nervosa. Eur Eat Disord Rev.

[47] Serpell L, Treasure J, Teasdale J, Sullivan V (1999). Anorexia nervosa: friend or foe?. Int J Eat Disord.

[48] Surgenor LJ, Plumridge EW, Horn J (2003). “Knowing one’s self” anorexic: implications for therapeutic practice. Int J Eat Disord.

[49] Offord A, Turner H, Cooper M (2006). Adolescent inpatient treatment for anorexia nervosa: a qualitative study exploring young adult’s retrospective views of treatment and discharge. Eur Eat Disord.

[50] Bulik CM (2014). The challenges of treating anorexia nervosa. Lancet.

