# Supplementary material for: Self-esteem as a catalyst for change in adolescent inpatients with anorexia nervosa: a pilot randomised controlled trial
Source: Eat Weight Disord. 2021 Mar 13;27(1):189–98. doi: 10.1007/s40519-021-01161-0 (PMC8860801; doi:10.1007/s40519-021-01161-0)
Supplement: Supplementary file 1 — Supplementary file1 (DOCX 73 KB) [file 40519_2021_1161_MOESM1_ESM.docx]

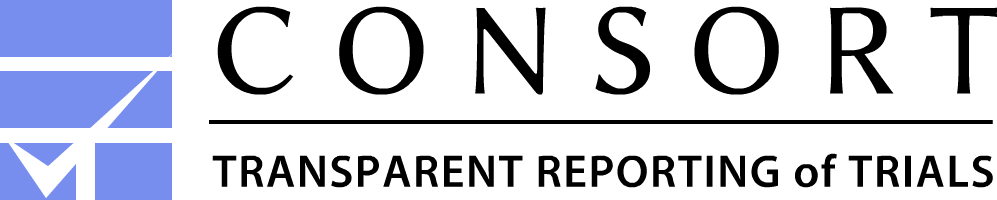


**CONSORT 2010 Flow Diagram**

Assessed for eligibility (n=86)

Excluded (n=36)

*Not meeting inclusion criteria (n=8)*

*Declined to participate (n=28)*

Randomized (n=50)

## Allocation

Allocated to control (n=25)

Allocated to intervention (n=25)

## Follow-Up

Discontinued intervention (give reasons) (n=10)

*Dropped out prior to T1 (n=2)*

1 – Discharged against medical advice prior to completion of T1

1 – Withdrew consent

*Dropped out prior to T2 (n=2)*

2 – Withdrew consent

*Dropped out prior to T3 (n=6)*

6 – Discharged prior to completion of T3

Completed Study to T2 (n=21)

Completed Study to T3 (n=15)

Dropped out of study (give reasons) (n=11)

*Dropped out prior to T1 (n=2)*

2 - Discharged prior to completion of T1

*Dropped out prior to T2 (n=3)*

3 – Discharged prior to completion of T2

*Dropped out prior to T3 (n=6)*

6 – Discharged prior to completion of T3

Completed Study to T2 (n=20)

Completed Study to T3 (n=14)

Analysis for T2 outcomes (n=21)
Excluded from analysis (give reasons) (n= 4)

Did not have data at all time points

Analysis for T3 outcomes (n=15)

Excluded from analysis (give reasons) (n=10)

Did not have data for all time points

## Analysis

Analysis for T2 outcomes (n=20)
Excluded from analysis (give reasons) (n=5)

Did not have data at all time points

Analysis for T3 outcomes (n=14)
Excluded from analysis (give reasons) (n=11)

Did not have data at all time points
